# Discovery of conjoined charge density waves in the kagome superconductor CsV_3_Sb_5_

**DOI:** 10.1038/s41467-022-33995-2

**Published:** 2022-10-26

**Authors:** Haoxiang Li, G. Fabbris, A. H. Said, J. P. Sun, Yu-Xiao Jiang, J.-X. Yin, Yun-Yi Pai, Sangmoon Yoon, Andrew R. Lupini, C. S. Nelson, Q. W. Yin, C. S. Gong, Z. J. Tu, H. C. Lei, J.-G. Cheng, M. Z. Hasan, Ziqiang Wang, Binghai Yan, R. Thomale, H. N. Lee, H. Miao

**Affiliations:** 1 Materials Science and Technology Division, Oak Ridge National Laboratory, Oak Ridge, TN 37831 USA; 2 Advanced Photon Source, Argonne National Laboratory, Argonne, IL 60439 USA; 3 Beijing National Laboratory for Condensed Matter Physics and Institute of Physics, Chinese Academy of Sciences, Beijing, 100190 China; 4 School of Physical Sciences, University of Chinese Academy of Sciences, Beijing, 100190 China; 5 Laboratory for Topological Quantum Matter and Advanced Spectroscopy (B7), Department of Physics, Princeton University, Princeton, NJ 08544 USA; 6 Laboratory for Quantum Emergence, Department of Physics, Southern University of Science and Technology, Shenzhen, Guangdong 518055 China; 7 Center for Nanophase Materials Sciences, Oak Ridge National Laboratory, Oak Ridge, TN 37831 USA; 8 National Synchrotron Light Source II, Brookhaven National Laboratory, Upton, NY 11973 USA; 9 Department of Physics and Beijing Key Laboratory of Opto-Electronic Functional Materials and Microdevices, Renmin University of China, Beijing, 100872 China; 10 Department of Physics, Boston College, Chestnut Hill, MA 02467 USA; 11 Department of Condensed Matter Physics, Weizmann Institute of Science, Rehovot, 7610001 Israel; 12 Institute for Theoretical Physics, University of Würzburg, Am Hubland, D-97074 Würzburg, Germany; 13 Present Address: Advanced Materials Thrust, The Hong Kong University of Science and Technology (Guangzhou), Guangzhou, Guangdong 511453 China; 14 Present Address: Department of Physics, Gachon University, Seongnam, 13120 Republic of Korea

**Keywords:** Electronic properties and materials, Phase transitions and critical phenomena

## Abstract

The electronic instabilities in CsV_3_Sb_5_ are believed to originate from the V 3*d*-electrons on the kagome plane, however the role of Sb 5*p*-electrons for 3-dimensional orders is largely unexplored. Here, using resonant tender X-ray scattering and high-pressure X-ray scattering, we report a rare realization of conjoined charge density waves (CDWs) in CsV_3_Sb_5_, where a 2 × 2 × 1 CDW in the kagome sublattice and a Sb 5*p*-electron assisted 2 × 2 × 2 CDW coexist. At ambient pressure, we discover a resonant enhancement on Sb *L*_1_-edge (2*s*→5*p*) at the 2 × 2 × 2 CDW wavevectors. The resonance, however, is absent at the 2 × 2 × 1 CDW wavevectors. Applying hydrostatic pressure, CDW transition temperatures are separated, where the 2 × 2 × 2 CDW emerges 4 K above the 2 × 2 × 1 CDW at 1 GPa. These observations demonstrate that symmetry-breaking phases in CsV_3_Sb_5_ go beyond the minimal framework of kagome electronic bands near van Hove filling.

## Abstract (web summary)

The nature of unconventional charge density wave in kagome metals is currently under intense debate. Here the authors report the coexistence of the 2 × 2 × 1 charge density wave in the kagome sublattice and the Sb 5p-electron assisted 2 × 2 × 2 charge density waves in CsV_3_Sb_5_.

## Introduction

**Fig. 1:**
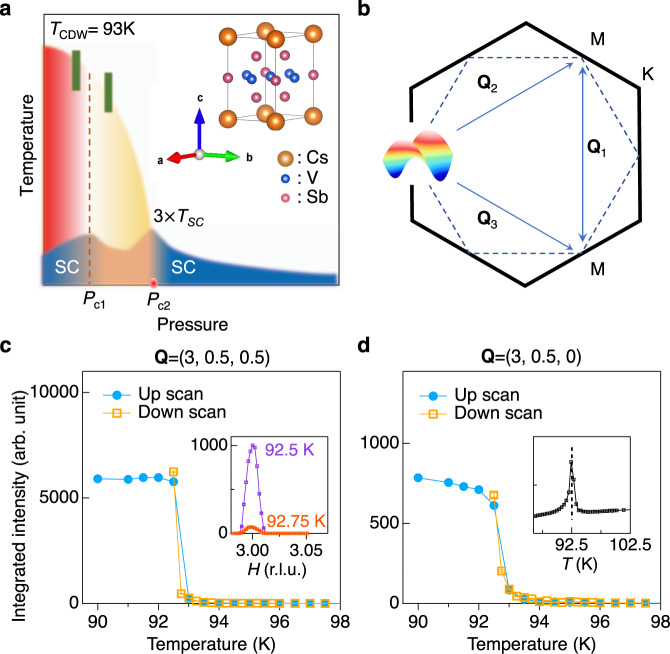
Charge density wave in CsV_3_Sb_5_. **a** *T-P* phase diagram and crystal structure of CsV_3_Sb_5_ (space group P6 = mmm, no. 191). The phase diagram is separated by two characteristic pressures, *P*_c1_ and *P*_c2_. The curvature of resistivity at *T*_CDW_ changes sign at *P*_c1_. At the same pressure, superconductivity reaches its first peak. The green markers indicate pressures where the high-pressure X-ray diffraction measurement was performed (Fig. 3). **b** A schematic of van Hove singularities at the M point of the hexagonal Brillouin zone. Theoretically, the van Hove filling induce Fermi surface instabilities with three nesting wavevector **Q**_1,2,3_. Integrated CDW intensity vs temperature measured at **Q**_**CDW**_ = (3, 0.5 0.5) (**c**) and (3, 0.5, 0) (**d**) using meV-resolution elastic X-ray scattering. A sharp jump of the order parameter at the CDW transition indicates a weak first order phase transition and is consistent with nuclear magnetic resonance measurement^41^. The intensity jump is much stronger in the out-of-plane CDW peak at (3, 0.5, 0.5) compared to the in-plane one at (3.5, 0.5, 0). The inset of **c** shows the CDW peak intensity measured at 92.5 K and 92.75 K. The specific heat data shown in the inset of **d** reveal a sharp transition at 92.5 K, consistent with the diffraction data. The error bars in **c**, **d** represent 1-standard deviation assuming Poisson counting statistics.

Charge density waves (CDWs), a translational symmetry-breaking electronic fluid state, are in the spotlight to unravel intertwined quantum materials. This includes cuprate high-*T*_C_ superconductors^1–3^, Moiré superlattices^4^, and nonmagnetic kagome metals where the CDW emerges as a leading electronic instability near van Hove filling^5–12^. Intriguingly, due to the sublattice interference on the geometrically frustrated triangle network, CDWs are predicted to potentially feature finite angular momentum or time-reversal symmetry breaking^7–12^. Recently, an exotic CDW in combination with superconductivity (SC) has been discovered in a kagome superconductor family *A*V_3_Sb_5_ (*A* = K, Rb, Cs) (Fig. 1a)^13–25^. At ambient pressure, a CDW in *A*V_3_Sb_5_ sets in between 78 K and 103 K. Electronic nematicity and time-reversal symmetry breaking that are potentially related to the CDW phase have been observed^15,16,22,24,25^. Below *T*_SC_~3 K, SC emerges and displays an unconventional pair-density modulation in real space^23^.

While it is apparent that the spatial symmetry breaking plays a key role in describing the exotic electronic phases in CsV_3_Sb_5_, the origin of CDW and its relationship with electronic nematicity and SC are yet to be discovered. Like the conventional vs. unconventional paradigm for superconducting pairing, a CDW can be mediated by phonons or electronic interactions, which is challenging to discriminate. This particularly applies to electronic kagome bands, where the 3*d*-van Hove singularities at the M point imply particle-hole fluctuations favoring a 2 × 2 × 1 CDW that preserves the *C*_6_ rotational symmetry (Fig. 1b)^5–9^. In experimental studies, the CDW in kagome metals appears to be 3-dimensional (3D) with a 2 × 2 × 2 superstructure^17,18^, indicating a non-trivial interlayer coupling that possibly breaks *C*_6_ to *C*_2_^11,12,26^. Still, a Landau theory analysis shows that the 2 × 2 × 2 CDW alone is incompatible with the first order phase transition (Fig. 1c, d), which could point to a novel coexistence of 2 × 2 × 1 and 2 × 2 × 2 CDWs^11,12^. Turning to the superconducting phase, the intimate correlation between CDW and SC extends to the temperature (*T*) *vs* pressure (*P*) phase diagram^19–21^. As depicted in Fig. 1a, the CDW transition temperature, *T*_CDW_, monotonically decreases and eventually vanishes at *P*_c2_. Near an intermediate pressure *P*_c1_~0.7 GPa, the curvature of the resistivity at *T*_CDW_ changes sign. Interestingly, SC also shows two turning points at *P*_c1_ and *P*_c2_ and forms a double-dome structure. In this letter, we use advanced X-ray scattering to demonstrate a rare realization of conjoined CDWs in CsV_3_Sb_5_. The conjoined CDWs transcend the phenomenology that could be derived from a sole V kagome sublattice near van Hove filling and provides spatial symmetry constraints to understand the enlarged complexity of novel symmetry-breaking phases in CsV_3_Sb_5_.

## Results

**Fig. 2:**
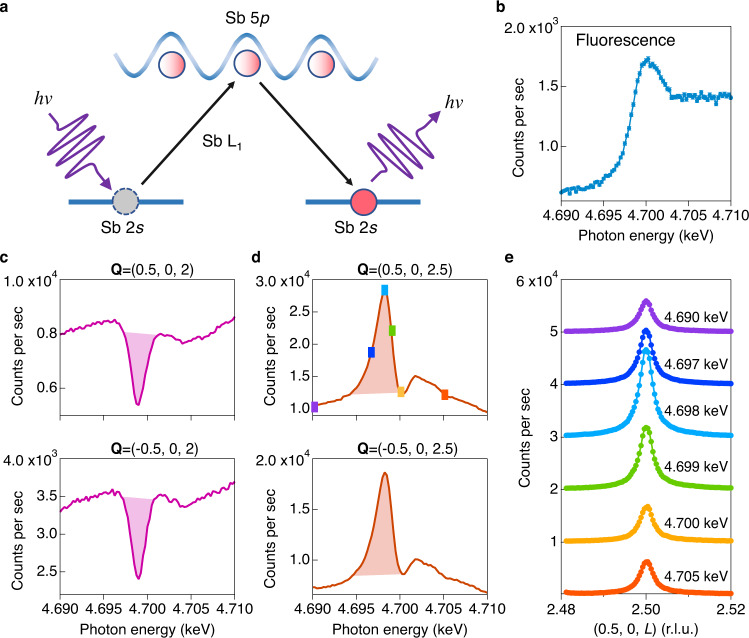
Sb *L*_1_-edge REXS reveals conjoined CDWs in CsV_3_Sb_5_. **a** Schematic of the REXS process. The Sb 2*s* to 5*p* transition is allowed by the dipole selection rule. **b** X-ray fluorescence near the Sb *L*_1_-edge (4.7 keV). **c**, **d** Photon energy scans at the ${{{{{{\bf{Q}}}}}}}_{{{{{{\rm{CDW}}}}}}}^{2\times 2\times 1}=(\pm\!0.5,\,0,\,2)$ and ${{{{{{\bf{Q}}}}}}}_{{{{{{\rm{CDW}}}}}}}^{2\times 2\times 2}=(\pm\!0.5,\,0,\,2.5)$ taken at *T* = 10 K. Resonant peaks in the energy scan of the 2 × 2 × 2 CDW demonstrate a Sb 5*p*-electron assisted CDW. This resonance can be directly observed in energy-dependent L-scans shown in **e**. The error bars in **b** represent 1-standard deviation assuming Poisson counting statistics.

We start by examining the electronic origin of this CDW using resonant elastic X-ray scattering (REXS). As depicted in Fig. 2a, by tuning the photon energy to atomic absorption edges, orbital-resolved valence electrons that are involved in the CDW will be enhanced^3^. For instance, in the cuprate high-*T*_c_ superconductors, CDW intensity resonates at both O *K* edge (1*s*→2*p*)^27^ and Cu *L*_3_ edge (2*p*→3*d*)^28,29^, reflecting the *d*-*p* hybridized wave function. In CsV_3_Sb_5_, while the V *L*_3_ edge (*hν* = 0.512 keV) is ideal to probe the 3*d*-electron contributions for the CDW, the low-photon energy prevents its access to the CDW wavevectors. Instead, we choose the Sb *L*_1_ edge (2*s*→5*p*, *hν* = 4.7 keV) to enhance the 5*p*-electrons near the Fermi level (see Supplementary Fig. 2 for REXS at other edges). At the Sb *L*_1_ edge, both ${{{{{{\bf{Q}}}}}}}_{{{\rm{CDW}}}}^{2\times 2\times 1}$ with *L* = *integer* and ${{{{{{\bf{Q}}}}}}}_{{{\rm{CDW}}}}^{2\times 2\times 2}$ with *L* = *half-integer* can be reached^18^. Moreover, since Sb 5*p*_*z*_-electrons form a large Fermi surface and directly contribute to the coupling between adjacent kagome layers, the Sb *L*_1_-edge resonance can potentially be used to distinguish the 2 × 2 × 1 CDW in the kagome sublattice and the interlayer coupled 2 × 2 × 2 CDW.

Our main observations at the Sb *L*_1_ edge are summarized in Fig. 2b–e. All REXS data were collected in the reflection geometry at *T* = 10 K (see Supplementary Fig. 1). The Sb *L*_1_ edge is identified around *hν* = 4.7 keV in the fluorescence scan shown in Fig. 2b. Figure 2c and d shows energy scans at fixed ${{{{{{\bf{Q}}}}}}}_{{{\rm{CDW}}}}^{2\times 2\times 1}=(\pm\!0.5,\,0,\,2)$ and ${{{{{{\bf{Q}}}}}}}_{{{\rm{CDW}}}}^{2\times 2\times 2}=(\pm\!0.5,\,0,\,2.5)$ (see data at more **Q** points in Fig. S1). Interestingly, we find a strong dip in scans at 2 × 2 × 1 wavevectors, which is in stark contrast to scans at 2 × 2 × 2 wavevectors that show large resonant enhancement slightly below the Sb *L*_1_ edge. The dramatically different resonant response between ${{{{{{\bf{Q}}}}}}}_{{{{{{\rm{CDW}}}}}}}^{2\times 2\times 1}$ and ${{{{{{\bf{Q}}}}}}}_{{{{{{\rm{CDW}}}}}}}^{2\times 2\times 2}$ demonstrates different electronic origins of the 2 × 2 × 1 and 2 × 2 × 2 CDWs in CsV_3_Sb_5_, where the Sb 5*p* valence electrons are only involved in the formation of the 2 × 2 × 2 CDW. An immediate consequence of two CDW order parameters is that the order parameter interference allows cubic free-energy terms in the Landau theory analysis^11,12^ and hence naturally explains the puzzling weak first order CDW transition in CsV_3_Sb_5_.

**Fig. 3:**
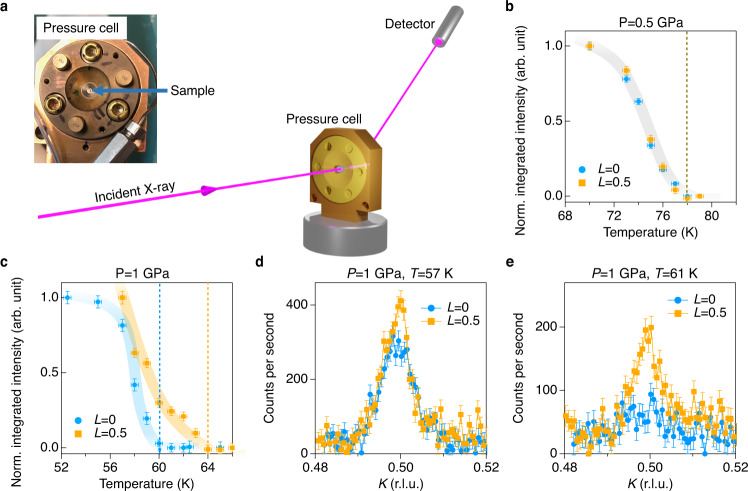
Evolution of conjoined CDWs under pressure. **a** The experimental geometry of high-pressure X-ray diffraction. **b**, **c** Normalized integrated CDW intensity vs temperature taken at ${{{{{{\bf{Q}}}}}}}_{{{{{{\rm{CDW}}}}}}}^{2\times 2\times 2}$=(3, 0.5, 0.5) and ${{{{{{\bf{Q}}}}}}}_{{{{{{\rm{CDW}}}}}}}^{2\times 2\times 1}$=(3, 0.5, 0) at *P* = 0.5 GPa and 1 GPa. Dashed lines mark the onset temperature of the 2 × 2 × 2 CDW (yellow) and 2 × 2 × 1 CDW (blue)_._ The intensity in **b** and **c** is normalized by the intensity at the lowest temperature in each curve. The high-pressure X-ray scattering were taken at 20 keV in a transmission geometry. The shadings in **b** and **c** are guides to the eye. **d**, **e** Direct comparison of the CDW peak intensity of ${{{{{{\bf{Q}}}}}}}_{{{{{{\rm{CDW}}}}}}}^{2\times 2\times 2}$ = (3, 0.5, 0.5) and ${{{{{{\bf{Q}}}}}}}_{{{{{{\rm{CDW}}}}}}}^{2\times 2\times 1}$ = (3, 0.5, 0) under 1 GPa. The measurements are taken at *T* = 57 K and 61 K. which is below and above the onset temperature (*T* = 60 K) of the 2 × 2 × 1 CDW (L = 0) under 1  GPa (blue dashed line in **c**). The temperature was determined by the thermal diode reading on the sample stage, which is stable on the level of 0.1 K. We estimated the upper limit of temperature error based on the *T*-dependent order parameter measurement shown in Fig. 1c of the main text, which is less than 0.3 K. The vertical error bars in **b–e** represent 1-standard deviation assuming Poisson counting statistics.

Having established the two conjoined CDWs with different charge contributions, we examine the CDW evolution under pressure. The experimental geometry of high-pressure diffraction is depicted in Fig. 3a. Figure 3b shows the integrated CDW intensity at *P* = 0.5 GPa < *P*_c1_ (the green rectangles in Fig. 1a mark the location of the measurement in the *T–P* phase diagram). Consistent with the ambient pressure measurement, the CDW peaks at ${{{{{{\bf{Q}}}}}}}_{{{{{{\rm{CDW}}}}}}}^{2\times 2\times 2}$ and ${{{{{{\bf{Q}}}}}}}_{{{{{{\rm{CDW}}}}}}}^{2\times 2\times 1}$ emerge at the same temperature around 77 K. Upon increasing pressure up to *P* = 1 GPa> *P*_c1_, however, the degeneracy of the conjoined CDWs is lifted with a 4 K gap between ${T}_{{{{{{\rm{CDW}}}}}}}^{2\times 2\times 2}$ and ${T}_{{{{{{\rm{CDW}}}}}}}^{2\times 2\times 1}$ (Fig. 3c). Figure 3d and e compares the 2 × 2 × 1 and 2 × 2 × 2 CDW intensities at 57 K and 61 K, respectively. The absence of the 2 × 2 × 1 CDW peak at 61 K provides independent evidence for two CDW order parameters in CsV_3_Sb_5_. Unexpectedly, the 2 × 2 × 2 CDW peak precedes the 2 × 2 × 1 peak at *P* > *P*_c1,_ casting doubt on scenarios based on a simple stacking of 2 × 2 × 1 superlattices with a π-phase shift, as this should lead to a higher ${T}_{{{{{{\rm{CDW}}}}}}}^{2\times 2\times 1}$ compared to ${T}_{{{{{{\rm{CDW}}}}}}}^{2\times 2\times 2}$. This observation further supports two CDW order parameters in CsV_3_Sb_5_ with different electronic origins. Indeed, our observation is consistent with a Landau theory analysis that showed two CDW transitions in the presence of order parameter couplings^12^. The separation of the conjoined CDW coincides with the superconducting crossover region in the *T* vs *P* phase diagram shown in Fig. 1a (orange shaded area). This observation suggests that the 2 × 2 × 1 CDW in the kagome sublattice is more closely related to the first superconducting dome, whereas the 5*p*-assisted 2 × 2 × 2 CDW has more impact on the second superconducting dome.

The observation of conjoined CDWs in CsV_3_Sb_5_ may also resemble the intertwined cuprate high-*T*_c_ superconductors, where under magnetic field^30,31^ or unidirectional pressure^32^ a short-ranged CDW broadly peak at *L* = *half-integer* coexists with a long-ranged CDW at *L* = *integer*. It should be noted, however, that the two conjoined CDWs in CsV_3_Sb_5_ are both long-range ordered with similar correlation length. This observation suggests entangled charge and lattice instabilities in CsV_3_Sb_5_ rather than the intertwined charge and spin correlations that are widely believed to be crucial for the cuprate high-*T*_c_ superconductors^1^.

**Fig. 4:**
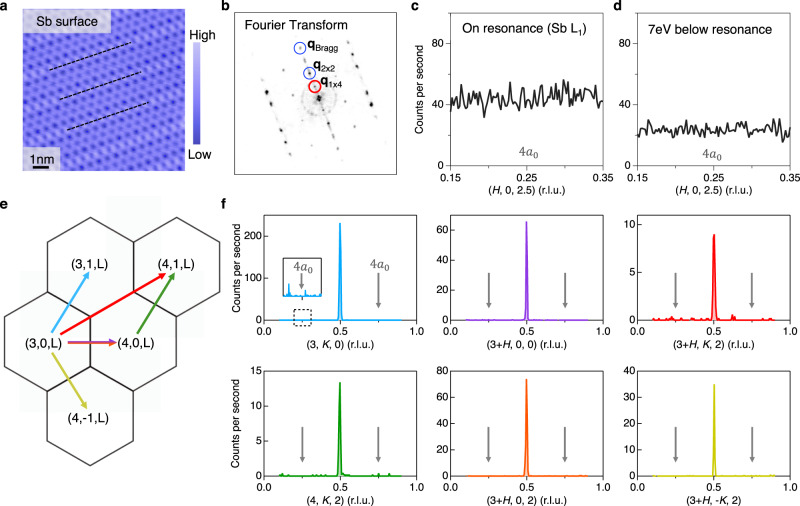
Absence of 1 × 4 superlattice peak in the X-ray diffraction measurement of bulk CsV_3_Sb_5_. **a** Topographic image of a clean Sb surface (*V* = −100 mV, *I* = 0.5 nA). **b** Fourier transform of the topography showing Bragg peaks and charge ordering vector peaks. The in-plane 2 × 2 CDW, 1 × 4 charge order and Bragg peaks are highlighted by circles. **c**, **d** On-resonance (Sb L_1_ edge, Fig. 2b) and under-resonance X-ray diffraction measurement around **Q** = (0.25, 0, 2.5) at *T* = 10 K. **e** Schematics indicating the scattering trajectories for scans shown in **f**. meV-resolution hard X-ray diffraction measurements shown in **f** were performed at 10 K and covered all in-plane high symmetry directions with zero and finite *L* components. The bulk CDW peaks can be clearly resolved in all scans in **f**, but there is no peak feature at *q* = 0.25 in neither the resonance measurements (**c**, **d**) nor the high-precision hard X-ray measurement (**f**).

Finally, we investigate the unidirectional 1 × 4 superlattice (4a_0_ phase) that was originally observed by scanning tunneling microscope/spectroscopy on the Sb surface^22^. The 4a_0_ phase emerges below an intermediate temperature T^*^ < 60 K and has been proposed as a possible analogy to the stripe order in cuprates^1^ and the cascade ordering in twisted bilayer graphene^33^. Figure 4a shows the topographic image of the clean Sb surface. In agreement with previous studies^22^, the Fourier transformation of the topography, shown in Fig. 4b, reveals both surface 2 × 2 and 1 × 4 superlattice modulations. As highlighted by the black dashed lines in Fig. 4a and the red circle in Fig. 4b, both the topographic imaging and the Fourier transformation result confirm the existence of the 1 × 4 superlattice on the Sb surface. While the Fourier transformed 2 × 2 and 1 × 4 superlattice are of comparable intensities on the Sb surface, the 4a_0_ phase has been transparent to thermodynamic, Raman and X-ray diffraction measurements^13,18,34,35^. These observations indicate that the STM observed 4a_0_ phase that has a correlation length greater than 30 nm^22^ is either a pinning of short-ranged bulk state on the Sb surface or a pure surface effect. To examine the possible short-ranged bulk state, we enhance X-ray sensitivity by (i) enhancing the Sb 5*p* electron contributions at the Sb *L*_1_-edge (Fig. 2b); (ii) suppressing fluorescence background intensity below the Sb *L*_1_-edge (Fig. 2b); and (iii) suppressing inelastic background intensity using meV-energy-resolution X-ray diffraction (see detailed descriptions in Supplementary Note 4 and 5, and Supplementary Fig. 5 and 6). As we summarize in Fig. 4c–f, the 4a_0_ superlattice peak is absent in all these measurements. Based on experimental sensitivity (see discussions in Supplementary Note 4), we estimate that at 10 K, the diffraction intensity of the 4a_0_ phase, if present, is more than 4-order magnitude smaller than the 2 × 2 × 2 CDW that can break *C*_6_ to *C*_2_^11,12^. We therefore conclude that the 4a_0_ phase, if present in the bulk, is not responsible for electronic nematicity observed in various bulk probes^25,36,37^.

In summary, using resonant tender X-ray scattering and high-pressure X-ray scattering, we demonstrate the coexistence of the 2 × 2 × 1 CDW in the kagome sublattice and the 5*p*-electron assisted 2 × 2 × 2 CDW in CsV_3_Sb_5_. The observation of conjoined CDWs performatively goes beyond the minimal framework of kagome electronic bands near van Hove filling, and thus provides critical information to resolve the persisting puzzle of CsV_3_Sb_5_.

## Methods

### Sample preparation and characterizations

Single crystals of CsV_3_Sb_5_ were grown from Cs ingot (purity 99.9%), V powder (purity 99.9%) and Sb grains (purity 99.999%) using the self-flux method^38^. The mixture was put into an alumina crucible and sealed in a quartz ampoule under partial argon atmosphere (~ 0.2 atm at 1273 K). The sealed quartz ampoule was heated to 1273 K at 100 K/h and kept there for 24 h to ensure the homogeneity of melt. Then it was cooled down to 1173 K at 50 K/h and further to 973 K with 2 K/h. Finally, the ampoule was taken out from the furnace and put in a centrifuge upside down. The ampoule was centrifuged at 50 *g* for 20 s to separate CsV_3_Sb_5_ single crystals from the flux. Except sealing and heat treatment procedures, all other preparation processes were carried out in an argon-filled glove box in order to prevent the reaction of Cs with air and water. The obtained crystals have a typical size of 2 × 2 × 0.1 mm^3^. CDW transition is clearly observed in specific heat measurement as shown in the inset of Fig. 1d.

### Resonant elastic X-ray scattering

Resonant single crystal X-ray diffraction was performed at the 4-ID-D beamline of the Advanced Photon Source (APS), Argonne National Laboratory (ANL). The X-rays higher harmonics were suppressed using a Si mirror and by detuning the Si (111) monochromator. Diffraction was measured using a vertical scattering plane geometry and horizontally polarized (*σ*) X-rays. The incident intensity was monitored by a He filled ion chamber, while diffraction was collected using a Si-drift energy dispersive detector with ~200 eV energy resolution. The probed absorption edges are close in energy (4.1–5.5 keV); thus, the use of this detector is key to reject the fluorescence background. The sample temperature was controlled using a He closed cycle cryostat and oriented such that X-rays scattered from the (001) surface.

### High-pressure X-ray diffraction

High-pressure single crystal X-ray diffraction was also measured at the 4-ID-D beamline of the APS, ANL. High pressure was generated using a modified Merrill-Bassett-type diamond anvil cell^39^ fitted with a pair of Boehler-Almax anvils of 800 μm culet diameter. A stainless-steel gasket was indented to 70 μm and a sample chamber of 400 μm diameter was laser cut. 4:1 methanol:ethanol was used as pressure media. Diffraction was measured in the transmission geometry in which X-rays penetrate through both diamond anvils and sample. The sample was cut into ~80 × 80 × 40 μm^3^ and oriented such that the c-axis is parallel to the X-ray direction when *θ* = 0°. Temperature was controlled using a He closed cycle cryostat. During the measurement, a piece of Au foil is placed next to the sample in the high-pressure cell. Pressure was calibrated as a function of temperature using the Au lattice constant^40^ and controlled in-situ using a He gas membrane. X-ray energy of 20 keV was used to minimize the diamond anvil attenuation. The incident intensity was measured using a N_2_ filled ion chamber, and diffraction was collected using a NaI scintillator.

### meV-resolution hard X-ray diffraction

High-precision X-ray scattering measurements shown in Fig. 1c,d and Fig. 4f was taken at 30-ID-C (HERIX), where the highly monochromatic X-ray beam of incident energy *E*_i_ = 23.7 keV (*λ *= 0.5226 $\mathring{\rm A}$) was focused on the sample with a beam cross section of 35 × 15  μm^2^ (horizontal × vertical). The total energy resolution of the monochromatic X-ray beam and analyzer crystals was Δ*E*~1.5 meV (full width at half maximum). The measurements were performed in transmission geometry.

### Scanning tunneling microscopy

Single crystals with size up to 2 mm × 2 mm were cleaved mechanically in situ at 77 K in ultra-high vacuum conditions, and then immediately inserted into the microscope head, already at Helium-4 base temperature (4.2 K). Topographic images in this work were taken with the tunneling junction set up *V* = 100 mV and *I* = 0.05 nA for exploration of areas typically 400 nm × 400 nm. When we found atomically flat and defect-free areas, we took topographic images with the tunneling junction set up *V* = 100 mV and *I* = 0.5 nA to resolve the atomic lattice structure as demonstrated in the paper. Tunneling conductance spectra were obtained with an Ir/Pt tip using standard lock-in amplifier techniques with a lock-in frequency of 997 Hz and a junction set up of *V* = 50 mV and *I* = 0.5 nA, and a root-mean-square oscillation voltage of 0.3 mV. Tunneling conductance maps were obtained with a junction set up of *V* = 50 mV and *I* = 0.3 nA, and a root-mean-square oscillation voltage of 5 mV.

## Supplementary information

Supplementary info

Peer Review File

## Data Availability

The data that support the findings of this study are available from the corresponding author on reasonable request.
