# Supplementary material for: Discovery of conjoined charge density waves in the kagome superconductor CsV3Sb5
Source: Nat Commun. 2022 Oct 26;13:6348. doi: 10.1038/s41467-022-33995-2 (PMC9606281; doi:10.1038/s41467-022-33995-2)
Supplement: Supplementary file 1 — Supplementary info [file 41467_2022_33995_MOESM1_ESM.pdf]

Supplementary Information for Discovery of Conjoined Charge Density Waves in the Kagome Superconductor CsV_3Sb_5

Haoxiang Li[1,*,§], G. Fabbris[2], A. H. Said[2], J. P. Sun[3,4], Yu-Xiao Jiang[5], J.-X. Yin[6,*], Yun-Yi Pai[1], Sangmoon Yoon[1,¶], Andrew R. Lupini[7], C. S. Nelson[8], Q. W. Yin[9], C. S. Gong[9], Z. J. Tu[9], H. C. Lei[9], J.-G. Cheng[3,4], . M. Z. Hasan[5], Ziqiang Wang[11], Binghai Yan[12], R. Thomale[13], H. N. Lee[1], and H. Miao[1,*]

[1]*Materials Science and Technology Division, Oak Ridge National Laboratory, Oak Ridge, Tennessee 37831, USA*

[2]*Advanced Photon Source, Argonne National Laboratory, Argonne, Illinois 60439, USA*

[3]*Beijing National Laboratory for Condensed Matter Physics and Institute of Physics, Chinese Academy of Sciences, Beijing 100190, China*

[4]*School of Physical Sciences, University of Chinese Academy of Sciences, Beijing 100190, China*

[5]*Laboratory for Topological Quantum Matter and Advanced Spectroscopy (B7), Department of Physics, Princeton, New Jersey 08544, USA*

[6]*Laboratory for Quantum Emergence, department of physics, Southern University of Science and Technology, Shenzhen, Guangdong 518055, China*

[7]*Center for Nanophase Materials Sciences, Oak Ridge National Laboratory, Oak Ridge, Tennessee 37831, USA*

[8]*National Synchrotron Light Source II, Brookhaven National Laboratory, Upton, NY 11973, USA*

[9]*Department of Physics and Beijing Key Laboratory of Opto-Electronic Functional Materials and Microdevices, Renmin University of China, Beijing 100872, China*

[10]*Department of Physics, Boston College, Chestnut Hill, Massachusetts 02467, USA*

[11]*Department of Condensed Matter Physics, Weizmann Institute of Science, Rehovot 7610001, Israel*

[12]*Institute for Theoretical Physics, University of Würzburg, Am Hubland, D-97074 Würzburg, Germany*

[#]Correspondence should be addressed to: haoxiangli@ust.hk, yinjx@sustech.edu.cn, miaoh@ornl.gov.

[§]Present address: Advanced Materials Thrust, The Hong Kong University of Science and Technology (Guangzhou), Guangzhou, Guangdong 511400, China

[¶]Present address: Department of Physics, Gachon University, Seongnam 13120, Republic of Korea

Supplementary Note 1. Additional resonant elastic X-ray scattering

The resonant X-ray scattering measurement was performed in the reflection geometry, which is depicted in Supplementary Fig. 1a. The incident X-ray beam is monochromatic with energy tuned to the absorption edge. Panel b and c present energy scans around the Sb L_1 edge taken at **Q**=(±0.5, 0, 3) and (±0.5, 0, 3.5) respectively. This set of data taken on different **Q**s from Fig. 2 of the main text shows a consistent result, where resonant peaks appear at *L=integer* and dip feature present at *L=half integer*. Besides the Sb L_1 edge ($2s \rightarrow 5p$), we also examine the Sb L_2 edge ($2p_{1/2} \rightarrow 5d$, E=4.38 keV) and L_3 edge ($2p_{3/2} \rightarrow 5d$, E=4.13 keV). Since the unoccupied Sb $5d$ states are far away from the E_F, the fluorescence scans (Supplementary Fig. 2) don't show strong resonance. Similarly, the energy scans at Q_{CDW} are also absent of resonant enhancement. The humps shown in Supplementary Fig. 2**c** and 2**d** are due to multiple scattering as confirmed by azimuthal scans.

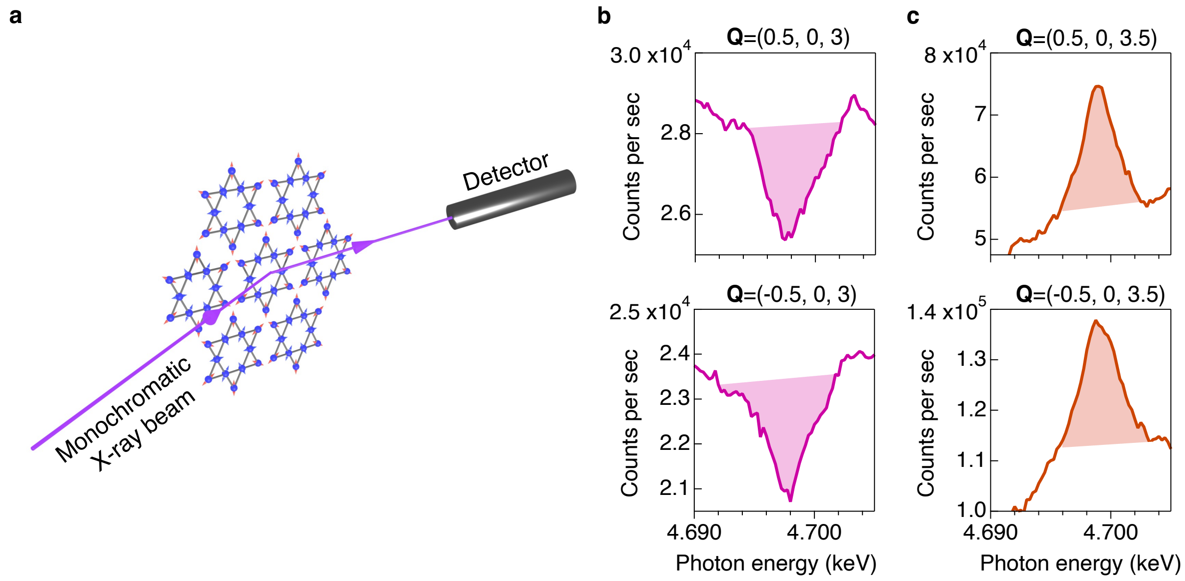

Supplementary Figure 1. Resonance data on Sb L_1 at more Q points on CsV_3Sb_5. a. Reflection geometry of the resonance tender X-ray diffraction measurement. **b,c**. energy scans around the Sb L_1 edge at **Q**=(±0.5, 0, 3) and (±0.5, 0, 3.5). These results taken at L=3 and 3.5 r.l.u. are consistent the results taken at L=2 and 2.5 r.l.u. shown in Fig. 2 of the main text.

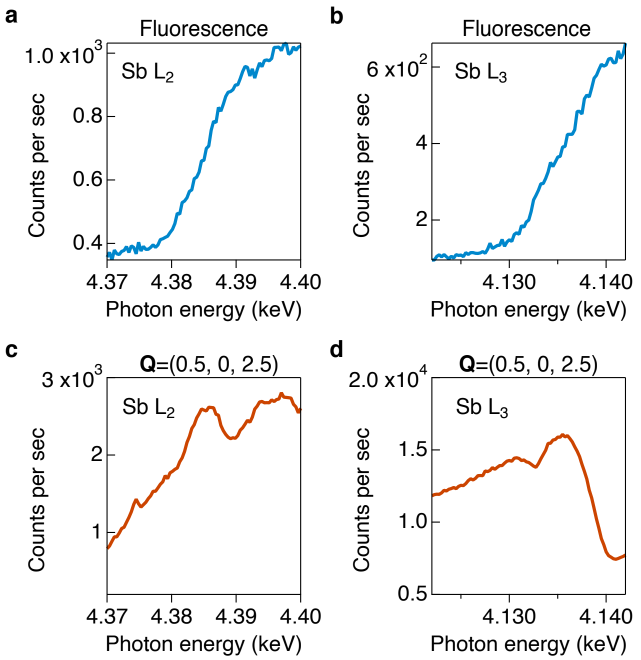

Supplementary Figure 2. X-ray fluorescence and resonant diffraction measurement taken at Sb L_2 edge (E=4.38 keV) and L_3 edge (E=4.13 keV).

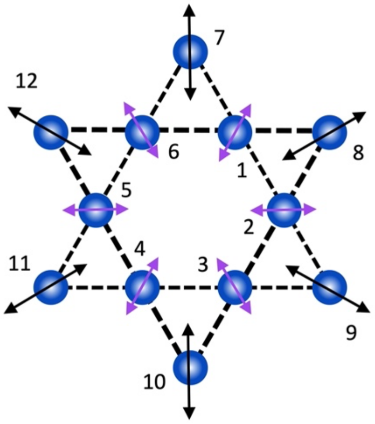

Supplementary Figure 3. SoD and inverse SoD lattice distortions of the V-kagome sublattice. Purple and black arrows indicate the lattice distortions are in opposite directions relative to the center of the hexagon.

Supplementary Note 2. A possible lattice response derived from the conjoined CDWs

The discovery of the conjoined CDWs raises question on the lattice responses. For instance, the emergence of 2×2×2 CDW usually induces superlattice peaks at both 2×2×1 and 2×2×2 CDW wavevectors. The totally different responses at Sb L_1-edge and pressure $P>P_{c1}$ between the 2×2×1 and 2×2×2 CDW wavevectors suggest that the 2×2×2 CDW displays a destructive interference at the 2×1 wavevectors. A similar case has been observed in the cuprate high-T_c superconductors, where the CDW peak is only observed at *L=half-integer*[1,2]

In this section we provide a possible solution for the lattice response of the conjoined CDWs. The diffraction intensity follows $I(\mathbf{Q})=|F(\mathbf{Q})|^2$, where $F(\mathbf{Q})$ is the scattering amplitude. For a crystalline material, $F(\mathbf{Q})$ can be formulated as:

$$F(Q) = \overbrace{\sum_{R_n} e^{iQ\cdot R_n}}^{lattice} \overbrace{\sum_j f_j(Q) e^{iQ\cdot r_j}}^{unit\ cell} = \delta_{Q=G} \overbrace{\sum_j f_j(Q) e^{iQ\cdot r_j}}^{unit\ cell} \quad (1)$$

where $\boldsymbol{R}_n$ and $\boldsymbol{G}$ are real and reciprocal lattice vectors, respectively. r_j is the jth atomic position in the unit cell. $\boldsymbol{Q}$ is the total momentum transfer and $f_j(\boldsymbol{Q})$ is the atomic form factor, which is derived from a Fourier transformation of local density of state[3]. Below T_{CDW}, the formation of CDW distorts the high-temperature structure and gives rise to superlattice peaks at $\boldsymbol{Q}$=$\boldsymbol{Q}_{CDW}$. Here we assume a simple star of David (SoD) and inverse SoD stacking:

$$F(Q) = \delta_{Q_{cdw}^{2\times2\times2}} \left\{ \overbrace{\sum_j f_j(Q) e^{iQ\cdot(r_j+\delta_j)}}^{SoD} + \overbrace{\sum_j f_j(Q) e^{iQ\cdot(r_j-\delta_j+c_0)}}^{inverse-SoD} \right\} \quad (2)$$

where c_0 is c-axis lattice constant in the normal state. δ_j is the lattice distortion in the CDW phase of the jth atom.

At L=half-integer:

$$F(Q) = \delta_{Q_{cdw}^{2\times2\times2}} \overbrace{\sum_j f_j(Q) e^{iQ\cdot r_j} (e^{iQ\cdot\delta_j} - e^{-iQ\cdot\delta_j})}^{2\times2\ superlattice}$$

$$= \delta_{Q_{cdw}^{2\times2\times2}} \overbrace{2i \sum_j f_j(Q) e^{iQ\cdot r_j} \sin(Q\cdot\delta_j)}^{2\times2\ superlattice}$$

$$\sim \delta_{Q_{cdw}^{2\times2\times2}} \overbrace{2i \sum_j f_j(Q) e^{iQ\cdot r_j} (Q\cdot\delta_j)}^{2\times2\ superlattice} \quad (Q\cdot\delta_j \ll 1) \quad (3)$$

At L=integer:

$$F(Q) = \delta_{Q_{cdw}^{2\times2\times2}} \overbrace{\sum_j f_j(Q) e^{iQ\cdot r_j} (e^{iQ\cdot\delta_j} + e^{-iQ\cdot\delta_j})}^{2\times2\ superlattice}$$

$$= \delta_{Q_{cdw}^{2\times2\times2}} \overbrace{2i \sum_j f_j(Q) e^{iQ\cdot r_j} \cos(Q\cdot\delta_j)}^{2\times2\ superlattice}$$

$$\sim -\delta_{Q_{cdw}^{2\times2\times2}} \overbrace{\sum_j f_j(Q) e^{iQ\cdot r_j} (Q\cdot\delta_j)^2}^{2\times2\ superlattice} \quad (Q\cdot\delta_j \ll 1) \quad (4)$$

Note $\overbrace{\sum_j f_j(Q) e^{iQ\cdot r_j}}^{2\times2\ superlattice} = 0$.

Supplementary Eq. (3) and (4) prove that under the condition of $(Q \cdot \delta_j \ll 1)$, the peak intensity at L=half-integer is $1/(Q \cdot \delta_j)$ stronger than at L=integer. Comparing the CDW peak with the nearby Bragg peak intensity, we find that $1/(Q \cdot \delta_j) \sim 10^3$.

If we consider only V-kagome sublattice, based on previous analysis[3], we find that:

$$F(Q) = \begin{cases} -4\pi\delta_{Q_{cdw}^{2\times2\times2}}(\delta + \epsilon)\ (Q = (0, 0.5, 0.5)) \\ 0\ (Q = (0, 0.5, 0)) \end{cases} \quad (5)$$

where δ and ϵ correspond to V^{1-6} and V^{7-12} distortions (Supplementary Fig. 3), respectively. Supplementary Eq. (5) assumes $|\delta|, |\epsilon| \ll a_0 = 5.4949$ Å, which is justified by previous XRD measurements, where the CDW superlattice peaks are about 3-order magnitude smaller than their nearby fundamental Bragg peaks.

Supplementary Note 3. 1×4 superlattice revealed by scanning tunnelling spectroscopy

Supplementary Fig. 4 shows the dI/dV images at ±20 meV, respectively. As highlighted between the black lines, these two spectroscopic maps reveal stripe like features with an intensity reversal, and the stripe features 1×4 charge modulations. Therefore, both the topographic imaging and spectroscopic imaging confirms the existence of 1×4 superlattice on the Sb surface.

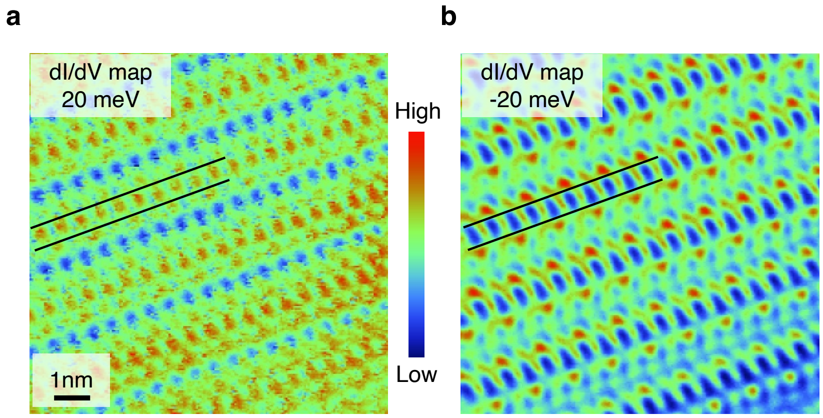

Supplementary Figure 4. Reversed bias peaks in scanning tunning spectroscopy. Panel **a** and **b** show atomically resolved dI/dV imaging for the same Sb surface at energy E=-20 meV and E=+20 meV, respectively.

Supplementary Note 4. Sensitivity of meV-XRD and resonant XRD measurements

The meV-resolution hard X-ray diffraction measurement was performed in the transmission geometry, where the experimental setup is shown in Supplementary Fig. 5**a**. The bandwidth of incident energy is 1 meV; this is done using a high-resolution monochromator (HRM) that consists of six silicon crystals[4-6]. The total energy resolution ΔE =1.5 meV is calibrated by fitting the elastic peak (inset of Supplementary Fig. 5a) to a pseudo-voigt function:

$$R(\omega) = (1-\alpha)\frac{I}{\sqrt{2\pi}\sigma}e^{-\frac{\omega}{2\sigma^2}} + \alpha\frac{I}{\pi}\frac{\Gamma}{\omega^2+\Gamma^2} \quad (1)$$

where the energy resolution is the full-width-at-half-maximum (FWHM). The extremely high energy resolution filters out background intensity[4] and false peaks appearing in conventional X-ray diffraction (see Supplementary Fig. 6), allowing experimental detection of static translational symmetry breaking even in the diffusive limit[1,2]. Supplementary Fig. 5**b** shows high-precision hard X-ray diffraction measurement of stripe ordered $La_{1.875}Ba_{0.125}CuO_4$ and optimally doped $La_{1.83}Sr_{0.17}CuO_4$. In these cuprate materials, the unidirectional $4a_0$ CDWs with correlation length, $\xi<20$ Å (compared with $\xi>300$ Å on the surface of CsV_3Sb_5) has been successfully detected[1,2], proving the high sensitivity of the meV-XRD measurement. As shown in Fig. 4 of the main text, the scattering intensity of the nearby CDW is on the order of 100s counts/sec with a background intensity less than 1 counts/sec. Therefore, based on this measurement, the $4a_0$ superlattice peak intensity, if present in the bulk, is more than 2~3 orders magnitude smaller than the CDW.

Similarly, we estimate our sensitivity near the Sb L_1 edge. As shown in Fig. 2e and Fig. 4c and d of the main text, the CDW peak intensity in the resonant X-ray scattering measurement is on the order of 10^4~10^5 counts/sec with a background intensity less than 50 counts/sec. A superlattice peak with intensity higher than 7 counts/sec (statistic error) should be distinguishable in our measurement. The absence of the $4a_0$ superlattice peak in our resonant X-ray scattering measurement demonstrates that the X-ray scattering intensity of the $4a_0$ phase (if it does exist in the bulk) is over 4-order magnitude smaller than the one of the 2×2×2 CDW that can potentially break rotation symmetry from C_6 to C_2 [3,7,8],

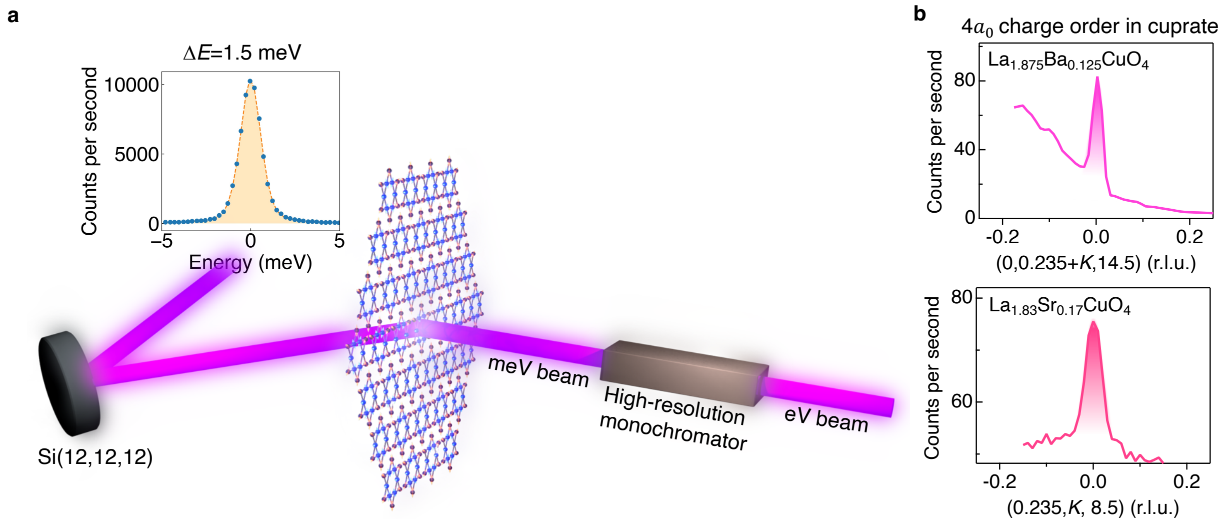

Supplementary Figure 5. **a**, Schematic of the meV-resolution hard X-ray diffraction measurement. The inset shows the inelastic X-ray scattering spectrum at **Q** = (3, 0, 0) on a CsV_3Sb_5 sample at T=10 K. The orange dashed line is a pseudo-voigt function fitting of the experimental data. The extracted energy resolution using the full width half maximum (FWHM) is ~1.5meV. **b**, Extracted high precision hard X-ray diffraction data of the charge ordered $La_{1.875}Ba_{0.125}CuO_4$.[1] and $La_{1.83}Sr_{0.17}CuO_4$[2].

Supplementary Note 5. Extrinsic XRD background

Supplementary Fig. 6 demonstrate the energy resolution effect in XRD measurement by comparing the XRD data with (purple) and without (orange) a MgO(440) analyzer. The experimental geometry is illustrated in Supplementary Fig. 6b. In the data taken without the analyzer (orange curve in Supplementary Fig. 6a), a sharp peak appears at 0.26 r.l.u., which intensity is much higher than that of the CDW peak at 0.5 r.l.u. Other broad background signals with comparable intensity to the CDW peak also appears in the data. These extrinsic features fully disappear in the data with the analyzer (purple curve in Supplementary Fig. 6a). It is important to note that multiple weak peaks can be identified in the purple curve, which could be falsely interpreted as superlattice structures at low temperature.

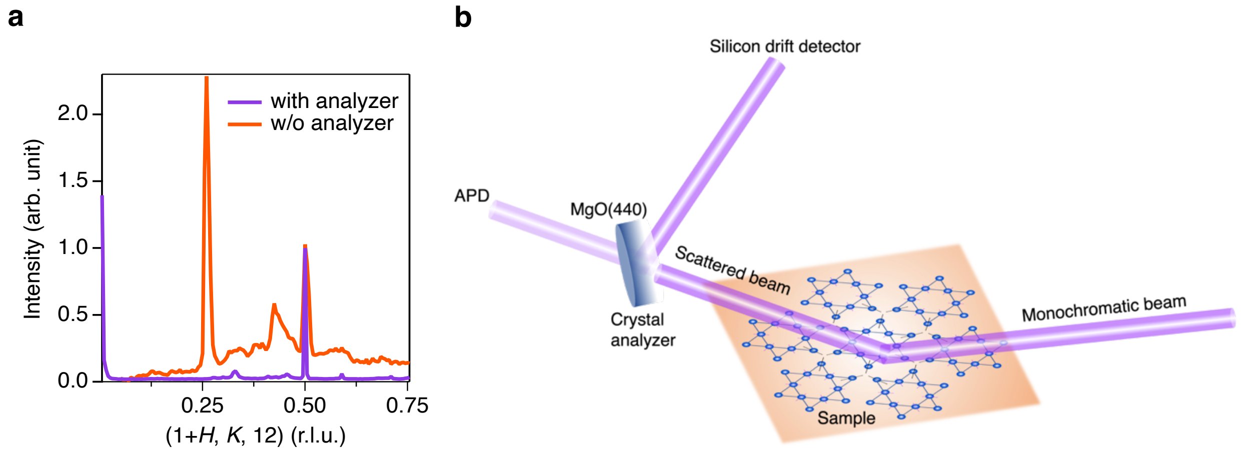

Supplementary Figure 6. Demonstration of energy resolution effect in XRD. **a**, XRD measurement of CsV_3Sb_5 at T=10 K, scanning from **Q**=(1,0,12) to (2,1,12). We compare the XRD spectra taken with (purple) and without (orange) a MgO(440) polarization analyzer that greatly reduce the scattered beam bandwidth (data taken at 4-ID beamline of NSLS2). The spectrum taken without analyzer shows a sharp peak at q~0.26 r.l.u., together with multiple broad background peaks near the CDW peak at q~0.5 r.l.u.. The q=0.26 r.l.u. peak and the broad background signal can effectively be filtered by the analyzer. **b**, Experimental geometry at the 4-ID beamline of NSLS2. Without the analyzer, the direct scattered beam is measured by the avalanche photodiode detector (APD). When a MgO(440) analyzer is placed before the APD to reduce the background scattering, the scattered beam from the analyzer is measured by a silicon drift detector.

Supplementary Note 6. Out-of-plane structural anomaly revealed by scanning transmission electron microscope (STEM)

Besides the *L=half-integer* CDW peak, superlattice peaks at *L=quarter-integer*, corresponding to a 2×2×4 CDW, have been reported in several X-ray studies[9-14]. However, existing experimental data showed that the 2×2×4 CDW varies strongly from sample to sample. For instance, Ortiz *et al.*[10] reported the 2×2×4 CDW peaks at T=15 K, which disappeared at 130 K. Chen *et al.*[11] showed that the *L=quarter-integer* peaks have no change from 2K to 190K, well beyond the CDW transition. Stahl *et al.*[12] reported that the 2×2×4 CDW only exists between 60K and 92K (T_{CDW}). Moreover, the K and Rb compounds and Sn doped CsV_3Sb_5 do not show the 2×2×4 superstructure[13]. In our study, while the selective resonant enhancement at *L=half-integer* is consistently observed in all our samples (5 different pieces), the 2×2×4 superlattice peaks were only observed in two of our samples. These results suggest that the 2×2×4 CDW does not play a key role for the conjoined CDWs. To find the origin of the out-of-plane structural anomaly in CsV_3Sb_5, we imaged the atomic structure along two different zone axes, i.e., [0001] and [1-100], using high-resolution high angle annular dark field scanning transmission electron microscopy (HAADF STEM). The data were collected on a sample that show 2×2×4 superstructure peaks.

Supplementary Fig. 7**a** shows a plan-view HAADF STEM image seen along the [0001] direction. Here, bright, and slightly fainter dots indicate Cs and Sb atomic columns, respectively. Since HAADF STEM provides scattering intensity that is approximately proportional to the square of the atomic number, the V atomic columns are not resolved in these images. This image shows that the in-plane lattice of CsV_3Sb_5 is highly periodic at room temperature, which is consistent with previous high-temperature STM results[15]. Supplementary Fig. 7**b** shows a cross-sectional HAADF STEM image viewed along the [1-100] zone axis. In this zone axis, the Cs atomic columns are darker than the Sb ones for this thin sample, because the atomic density of Cs is twice lower than that of Sb. Remarkably, the distance between V_3Sb_5 layers was significantly modulated in the c-axis, which is not expected in stoichiometric CsV_3Sb_5 samples. The interlayer distance was quantified by measuring the distance between Sb atomic columns across the Cs layers, demonstrating that the variation of lattice modulation is over 8 % (Supplementary Fig. 7**c**). This lattice modulation corroborates with the superlattice peak observed in the XRD measurement, and it could be attributed to several factors, such as an unconventional displacement of Cs atoms, related defects, or Cs-vacancies, but further characterizations are required to definitively characterize the origin of this structural modulation.

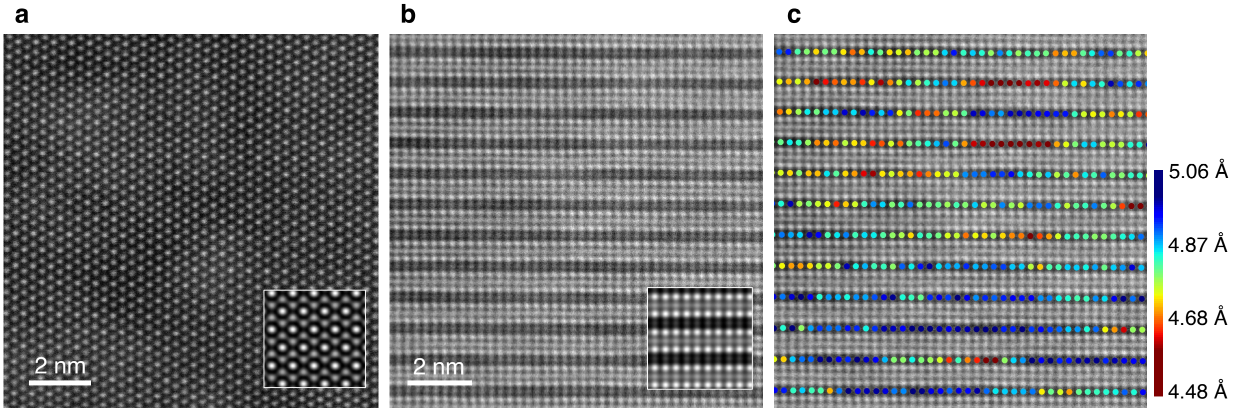

Supplementary Figure 7. Unconventional out-of-plane structural modulation of Cs layers in CsV_3Sb_5. High-resolution HAADF STEM image of CsV_3Sb_5 in the (**a**) plan-view and (**b**) cross-section geometries (measured at room temperature). Insets show the simulated HAADF STEM images of CsV_3Sb_5 in each zone axis. **c**. The map of the interlayer distance between V_3Sb_5 layers, where the distances are represented on the Cs atomic columns with a color code. The lattice of CsV_3Sb_5 is significantly modulated along the out-of-plane axis while it remains highly periodic in the in-plane directions.

References:

1. Miao, H. et al. Incommensurate phonon anomaly and the nature of charge density waves in cuprates, *Phys. Rev. X* **8**, 011008 (2018).
2. Miao, H. et al. Charge density waves in cuprate superconductors beyond the critical doping, *npj Quantum Mater.* **6**, 31 (2021).
3. Miao, H et al., Geometry of the charge density wave in kagome metal AV_3Sb_5. Phys. Rev. B 104, 195132 (2021).
4. Baron, A. Q. R., High-resolution inelastic X-Ray scattering II: scattering theory, harmonic phonons, and calculations. *Synchrotron Light Sources and Free-Electron Lasers* 1721–1757 (2016). doi:10.1007/978-3-319-14394-1_52.
5. Toellner, T. S., Alatas, A. and Said, A. H., Six-reflection meV-monochromator for synchrotron radiation. *J. Synchrotron Rad*. **18**, 605-611 (2011).
6. Said, A. H. et al. High-energy-resolution inelastic X-ray scattering spectrometer at beamline 30-ID of the Advanced Photon Source, *J. Synchrotron Rad.* **27**, 827 (2020).
7. Christensen, M. H. et al., Theory of the charge density wave in AV3Sb5 kagome metals, *Phys. Rev. B* **104**, 214513 (2021).
8. Park, T., Ye, M., and Balents, L., Electronic instabilities of kagome metals: Saddle points and Landau theory, *Phys. Rev. B* **104**, 035142 (2021).
9. Li, H. et al., Observation of Unconventional Charge Density Wave without Acoustic Phonon Anomaly in Kagome Superconductors AV_3Sb_5, *Phys. Rev. X* **11**, 031050 (2021).
10. Ortiz, B. R., et al. Fermi surface mapping and the nature of charge density wave order in the kagome superconductor CsV_3Sb_5, *Phys. Rev. X* **11**, 041030 (2021).
11. Chen, Q. et al. Charge density wave order and fluctuations above T_{CDW} and below superconducting Tc in the kagome metal CsV_3Sb_5. *Phys. Rev. Lett.* **129**, 056401 (2022).
12. Stahl, Q. *et al*., Temperature-driven reorganization of electronic order in CsV_3Sb_5. *Phys. Rev. B* **105**, 195136 (2022).
13. Oey, Y. M. *et al.*, Fermi level tuning and double-dome superconductivity in the kagome metal $CsV_3Sb_{5-x}Sn_x$. *Phys. Rev. Materials* **6**, L041801 (2022).
14. Xiao, Q. et al. Preprint at https://arxiv.org/abs/2201.05211
15. Zhao, H. et al. Cascade of correlated electron states in the kagome superconductor CsV_3Sb_5, *Nature* **599**, 216 (2021)
